# Potential Biomarkers for Radiation-Induced Renal Toxicity following ^177^Lu-Octreotate Administration in Mice

**DOI:** 10.1371/journal.pone.0136204

**Published:** 2015-08-19

**Authors:** Emil Schüler, Maria Larsson, Toshima Z. Parris, Martin E. Johansson, Khalil Helou, Eva Forssell-Aronsson

**Affiliations:** 1 Department of Radiation Physics, Institute of Clinical Sciences, Sahlgrenska Cancer Center, Sahlgrenska Academy at the University of Gothenburg, Sahlgrenska University Hospital, Gothenburg, Sweden; 2 Department of Oncology, Institute of Clinical Sciences, Sahlgrenska Cancer Center, Sahlgrenska Academy at the University of Gothenburg, Sahlgrenska University Hospital, Gothenburg, Sweden; 3 Department of Laboratory Medicine, Lund University, SUS Malmö, Malmö, Sweden; 4 Department of Medical Physics and Medical Bioengineering, Sahlgrenska University Hospital, Gothenburg, Sweden; Oxford Brookes University, UNITED KINGDOM

## Abstract

The kidneys are one of the main dose-limiting organs in peptide receptor radionuclide therapy and due to large inter-individual variations in renal toxicity, biomarkers are urgently needed in order to optimize therapy and reduce renal tissue damage. The aim of this study was to investigate the transcriptional, functional, and morphological effects on renal tissue after ^177^Lu-octreotate administration in normal mice, and to identify biomarkers for radiation induced renal toxicity.

**Methods:**

C57BL/6N mice were i.v. injected with 0, 30, 60, 90, 120, or 150 MBq ^177^Lu-octreotate (0, 16, 29, 40, 48, and 54 Gy to the kidneys). At 4, 8, and 12 months after administration, radiation-induced effects were evaluated in relation to (a) global transcriptional variations in kidney tissues, (b) morphological changes in the kidneys, (c) changes in white and red blood cell count as well as blood levels of urea, and (d) changes in renal function using ^99m^Tc-DTPA/^99m^Tc-DMSA scintigraphy.

**Results:**

In general, the highest number of differentially regulated transcripts was observed at 12 months after administration. The *Cdkn1a*, *C3*, *Dbp*, *Lcn2*, and *Per2* genes displayed a distinct dose-dependent regulation, with increased expression level with increasing absorbed dose. *Ifng*, *Tnf*, and *Il1B* were identified as primary up-stream regulators of the recurrently regulated transcripts. Furthermore, previously proposed biomarkers for kidney injury and radiation damage were also observed. The functional investigation revealed reduced excretion of ^99m^Tc-DTPA after 150 MBq, an increased uptake of ^99m^Tc-DMSA at all dose levels compared with the controls, and markedly increased urea level in blood after 150 MBq at 12 months.

**Conclusion:**

Distinct dose-response relationships were found for several of the regulated transcripts. The *Cdkn1a*, *Dbp*, *Lcn2*, and *Per2* genes are proposed as biomarkers for ^177^Lu-octreotate exposure of kidney. Correlations to functional and morphological effects further confirm applicability of these genes as markers of radiation damage in kidney tissue.

## Introduction

The development of biomarkers for clinical practice requires a detailed understanding of the disease under investigation [1]. After exposure to stressors, e.g. radiopharmaceuticals, induced kidney injury is the result of the relationship between, for example, cell dysfunction, cell death, proliferation, inflammation, and recovery. Therefore, these cellular processes need to be better understood in order to identify biomarkers indicative of radiation-induced kidney injury [2].

Therapy with radiolabeled somatostatin (SS) analogues has shown promising results concerning tumor regression, increased overall survival, and improved quality of life for patients with somatostatin receptor (SSTR) overexpressing neuroendocrine tumors [3, 4]. Uptake of radiolabeled SS analogues in normal tissues is generally lower than in tumor tissue [5, 6]. The highest normal tissue uptake takes place in the kidneys which is one of the main critical organs, beside bone marrow, for therapy [7–10]. There is a clear need to define biomarkers of radiation-induced kidney injury in order to avoid adverse effects during this type of therapy.

To assess the applicability of potential biomarkers, their relation to kidney function needs to be established including e.g. glomerular filtration rate (GFR), tubular reabsorption and secretion. Well-established methods to measure GFR include renal inulin clearance and total plasma clearance of ^51^Cr-EDTA [11]. However, repeated urine and blood sampling is cumbersome on mice after injection of ^51^Cr-EDTA. Instead scintigraphy might be an alternative to measure renal function [12]. ^99m^Tc-DTPA is exclusively excreted into the urine by glomerular filtration, without tubular secretion or reabsorption [13]. Tubular reabsorption and secretion is instead preferably measured by ^99m^Tc-DMSA scintigraphy [14]. The ^99m^Tc-DMSA uptake process is not entirely known, but seems to include both tubular secretion from blood and glomerular filtration followed by reabsorption [15]. A previous study indicated that ^99m^Tc-DMSA uptake is mediated by the megalin-cubilin endocytosis which is interesting because it mimics one of the uptake pathways of SS [16].

Upon kidney function disorders, alterations in blood levels of albumin, creatinine, urea, and cystatin C levels may be induced, alterations which might be used as biomarkers of kidney failure [13]. The most commonly used molecular biomarker for the assessment of renal function is serum creatinine. However, the usefulness of serum creatinine in renal injury assessment is probably limited because of a lack of power for the identification of early renal injury and dysfunction, as well as a strong dependence on muscle mass, age, sex, medications, and hydration status [2]. Instead, serum cystatin C has been proposed as marker of GFR and appears to be less dependent of age, sex, and muscle mass, which may indicate a more reliable marker [17]. Other biomarkers for kidney injury have also been proposed, such as KIM-1 and NGAL [18, 19]. However, their role as molecular biomarkers for radiation-induced kidney injury after radiopharmaceutical administration needs to be investigated.

The transcriptional response of normal tissues following radiopharmaceutical administration has previously been investigated following ^131^I, ^211^At, and ^177^Lu exposure [20–24]. It was found that the molecular response is highly dependent on both type of radionuclide used, as well as time after administration, absorbed dose, and dose rate. Concerning the kidneys, the molecular response 24 h after ^177^Lu-octreotate administration revealed strong dose-dependent association with metabolic and stress response related processes. Furthermore, recurrent regulation of e.g. *Havcr1* (KIM-1) and *Lcn2* (NGAL) were found, indicating induced kidney injury at these absorbed doses (up to 140 MBq of injected activity). These studies were short-term (3h-7d) and performed on Balb/c mice that are found more radiation sensitive compared with normal mice [24, 25].

In patients treated with ^177^Lu-octreotate, potential kidney toxicity occurs late after administration [26, 27]. To our knowledge, the molecular mechanisms behind late kidney toxicity have not been studied previously. In order to further our understanding of underlying mechanisms of late kidney toxicity long-term studies on transcriptional regulation after administration should be performed in normal mice, in combination with histological and functional examinations.

The aim of this study was to investigate the transcriptional, morphological and functional effects on renal tissue 4, 8 and 12 months after ^177^Lu-octreotate administration in normal mice, and to identify potential biomarkers for radiation induced renal toxicity.

## Materials and Methods

### Radiopharmaceuticals

Preparation of ^177^Lu-DOTA-Tyr^3^-octreotate (^177^Lu-octreotate) was performed according to the manufacturer’s instructions (I.D.B. Holland, Baarle-Nassau, Netherlands). The fraction of peptide bound ^177^Lu was higher than 99% in the final solution, as determined by instant thin layer chromatography.

^99m^Tc-DTPA (diethylene-triaminepenta-acetate) and ^99m^Tc-DMSA (dimercaptosuccinic acid) were acquired from Covidien (Millington, Dublin, Ireland), and preparation was performed according to the manufacturer’s instructions.

The activity of the syringes was measured before and after administration with a well-type ionization chamber (CRC-15R; Capintec, IA, USA) to determine the injected activity.

### Animal handling

C57BL/6N mice (5–6 weeks old) were acquired from Taconic (Hudson, USA). A total of 45 mice (n = 3 per group) were i.v. injected with 30, 60, 90, 120, and 150 MBq ^177^Lu-octreotate. Nine mice were injected with saline solution and used as age-matched controls. Mice were killed at 4, 8, or 12 months after injection by cardiac puncture under anesthesia (pentobarbitalnatrium, APL, Sweden) followed by blood sample collection and excision of kidneys. One kidney from each animal was subjected to transcriptional microarray analysis, whereas the remaining kidney was used for histological evaluation. Furthermore, 16 mice (n = 4 per group) were i.v. injected with 0, 30, 90, and 150 MBq ^177^Lu-octreotate and subjected to scintigraphic examination at 4, 8, and 12 months after administration.

This study was carried out in strict accordance with the recommendations on use of laboratory animals and all efforts were made to minimize suffering of the animals. The experimental protocol was approved by the Ethics Committee on Animal Experiments in Gothenburg, Sweden (permit number: 166–204).

### Dosimetry

The absorbed dose calculations to the kidneys were based on the Medical Internal Radiation Dose (MIRD) pamphlet 21 formalism [28]:

$$\bar{D}\left(r_{S},T_{D}\right)\;=\;\frac{\tilde{A}\left(r_{S},T_{D}\right)\sum_{i}E_{i}Y_{i}\phi \left(r_{T}\leftarrow r_{S},E_{i},T_{D}\right)}{M\left(r_{T},T_{D}\right)},$$

where $\tilde{A}(r_{S},T_{D})$ is the time integrated activity, *r*_*S*_ and *r*_*T*_ the source and target tissue, respectively, *T*_*D*_ is the dose-integration period, *E*_*i*_ is the energy of the i^th^ nuclear transition, *Y*_*i*_ is the number of i^th^ nuclear transitions per nuclear transformation, *ϕ*(*r*_*T*_←*r*_*S*,_ *E*_*i*,_ *T*_*D*_) is the absorbed fraction, and *M*(*r*_*T*,_ *T*_*D*_) is the mass of the target tissue. *r*_*S*_ was set to be the same as *r*_*T*_ in the calculations. Σ_*i*_ *E*_*i*_ *Y*_*i*_ was approximated to 147 keV, only including electrons emitted. The absorbed fraction, *ϕ*(*r*_*T*_←*r*_*S*,_ *E*_*i*,_ *T*_*D*_) was set to 0.93 [29]. $\tilde{A}(r_{S},T_{D})$ was based on biodistribution data on C57BL/6N mice after ^177^Lu-octreotate administration [30]. The absorbed dose was calculated to whole kidney.

### Transcriptional data processing

Genome-wide transcriptional analysis and data processing has been previously described [24, 31].

Homogenization of the tissue samples was performed with the Mikro-Dismembrator S ball mill (Sartorius Stedim Biotech) and total RNA extraction was performed with the RNeasy Lipid Tissue Mini Kit (Qiagen), according to the instructions of the manufacturer. The quality assessment of the samples was conducted through the NanoDrop ND-1000 and the RNA 6000 Nano LabChip Kit with Agilent 2100 Bioanalyzer (Agilent Technologies), with a RIN cut-off value of 6.

The RNA samples were processed at SCIBLU (Swegene Center for Integrative Biology at Lund University) using MouseRef-8 Whole-Genome Expression Beadchips (Illumina). The image acquisition and analysis were performed with the Illumina BeadArray Reader scanner and BeadScan 3.5.31.17122, respectively.

The web-based BioArray Software Environment (BASE) was used for data preprocessing and quantile normalization. Nexus Expression 3.0 (BioDiscovery, El Segundo, CA, USA) was used for further data analysis using log2-transformed, normalized expression values, and variance filter. Differential expression was determined using the Benjamini-Hochberg method to control the false discovery (FDR-corrected P value < 0.01). A log_2_-ratio > 0.58 were considered significant. Generated transcriptional profiles are available from the NCBI’s Gene Expression Omnibus (GEO accession number GSE54674).

Further analysis, including up-stream regulators and pathway analysis, was conducted using the IPA software (Ingenuity Systems, Redwood City, USA).

### Histological evaluation

The formalin fixed kidneys were processed by standard techniques for embedding in paraffin and sections (2 μm) were stained with haematoxylin-eosin. The evaluation of kidney histology was performed by a kidney pathologist in a blinded fashion.

### ^99m^Tc-DTPA and ^99m^Tc-DMSA scintigraphy

^99m^Tc-DTPA and ^99m^Tc-DMSA scintigraphy was performed at 4, 8, and 12 months after ^177^Lu-octreotate administration. A single headed gamma camera (ADACT 210, ADAC Laboratories A/S, Aalsborg, Denmark) equipped with medium energy parallel-hole collimator was used. Images were acquired with a 20% energy-window centered over the 140 keV photon peak, with an image matrix size of 256x256. For calibration, a syringe with known ^99m^Tc activity was included in each image.

About 55 MBq ^99m^Tc-DTPA was i.v. injected and posterior 1-minute images were obtained every fifth min between 2.5 and 32.5 min after injection. Two days later, 40 MBq ^99m^Tc-DMSA was i.v. injected and a posterior 3-minute image was acquired 3h after injection.

Image processing was performed with ImageJ software [32]. Regions-of-interest (ROIs) were outlined for each kidney at 10% of maximal pixel count level, for whole body and around the calibration syringe, and the total number of counts in each ROI was determined. The bladder content was determined at 27.5 min after ^99m^Tc-DTPA administration. The activity in the kidneys was calculated, assuming similar attenuation for the activity in the kidneys and the calibration syringe.

Statistical analyses with paired Student t-test were used, and p < 0.05 was regarded as statistically significant.

### Blood analyses

After cardiac puncture, blood was collected in a heparin (Orifarm, Odense, Denmark) coated needle. To assess blood cellular content, whole blood was analyzed by the Sysmex pocH-100i automated hematology system (Sysmex, Kobe, Japan), according to the manufacturer’s instructions.

Creatinine and urea levels in whole blood were analyzed using the Reflotron (Roche Diagnostics, Mannheim, Germany) according to the manufacturer’s instructions.

## Results

### Transcriptional response

The absorbed dose to the kidneys was estimated to 16, 29, 40, 48, and 54 Gy after injection of 30, 60, 90, 120, and 150 MBq ^177^Lu-octreotate.

**Fig 1:**
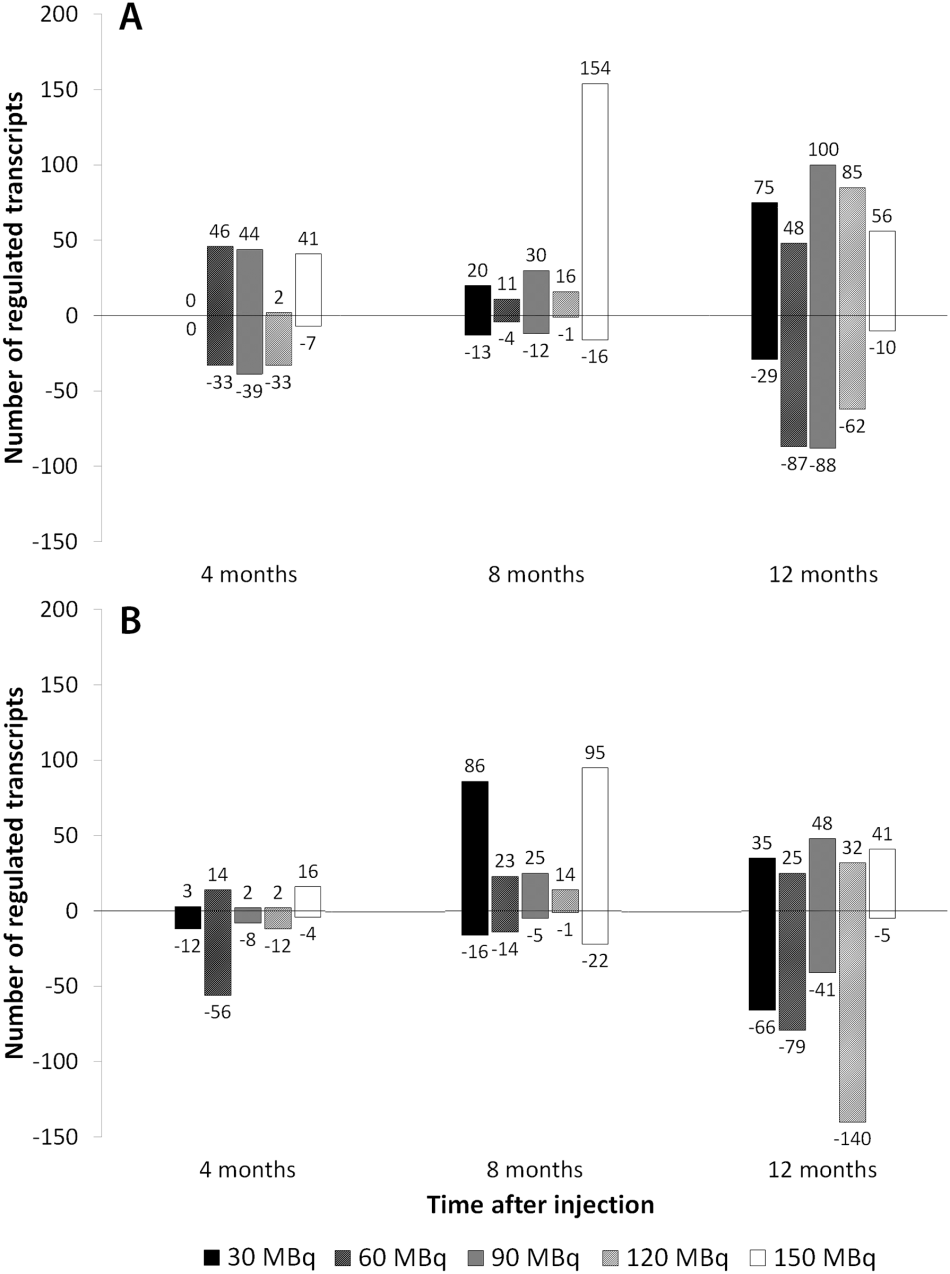
Number of differentially regulated transcripts. Number of differentially regulated transcripts in (a) kidney cortex and (b) kidney medulla 4, 8 and 12 months after administration of 30–150 MBq ^177^Lu-octreotate. Distribution of up- and down-regulated transcripts is indicated with positive and negative numbers, respectively

The number of differentially regulated transcripts ranged from 0 to 188 in kidney cortex, and from 10 to 172 in kidney medulla (Fig 1). A total of 34 and 32 of the differentially regulated transcripts in kidney cortex and medulla, respectively, have previously been suggested associated with kidney injury or radiation damage (Table 1), e.g. *Mmp2*, *Cdkn1a*, and *Ccnd1*. The strongest association with kidney damage-associated markers was found at 8 months for both tissues, with recurrent expression of e.g. *Cdkn1a*, *S100a6*, and *Lcn2*. At 4 and 8 months after injection, the highest number of kidney injury markers and/or radiation markers was found after 150 MBq. At 12 months, the strongest association was observed at 90 and 120 MBq in both tissues.

**Table 1:** Radiation responsive genes. Differentially regulated transcripts which have previously been proposed as biomarkers for kidney injury, radiation induced damage, and radiation biodosimetry [33–36]. The log_2_ratio is shown for each gene.

Time	Kidney Cortex						Kidney Medulla					
	Gene symbol	30 MBq	60 MBq	90 MBq	120 MBq	150 MBq	Gene symbol	30 MBq	60 MBq	90 MBq	120 MBq	150 MBq
**4 months**	*Bcat1* *			0.65		0.63	*Cyr61* *	0.75				
	*Egr1* *		1.2	1.3		1.2	*Egr1* *	1.7	1.1	1.1		1.2
	*Gdf15* *					0.72	*Igfbp3* *					-0.70
	*Hspa1a* *					0.80	***Mmp2*** *					1.0
	*Lcn2* *					1.0	*S100a6* *		-0.73			
	***Mmp2*** *					0.83	***Cdkn1a*** †		0.69	1.1	1.2	1.8
	***Cdkn1a*** †			1.2	1.49	2.0	***Fos*** †	0.91				
	*Ccng1* ‡					0.67	*Cxcl12* ‡		-0.99			
	*Gadd45g* ‡					1.5	*Gadd45g* ‡		-0.69		-0.64	1.2
	*Trp53inp1* ‡					0.70	*Tnfrsf21* ‡		-0.81			
**8 months**	*Akap12* *					0.63	*Anxa2* *					0.62
	***Anxa3*** *					0.88	***Anxa3*** *					0.78
	*Col3a1* *					0.65	*Col3a1* *					0.72
	*Col4a1* *					0.81	*Col4a1* *					0.66
	*Cxcl1* *					1.7	*Cxcl1* *					1.4
	*Cyr61* *					0.97	*Cyr61* *					0.71
	*Egr1* *					1.7	*Egr1* *		0.79	1.3		1.8
	*Gdf15* *				0.97	1.4	*Gdf15* *					1.2
	*Havcr1* *					1.6	*Havcr1* *				1.6	1.3
	*Hspa1a* *			0.61			*Hspa1a* *		0.79	1.0		
	***Icam1*** *					0.87	*Igfbp3* *					-0.77
	*Lcn2* *				1.8	2.0	*Lcn2* *		0.86		1.6	1.9
	*Lgals3* *					0.94	*Lgals3* *					0.87
	***Mmp2*** *				1.3	1.5	***Mmp2*** *				1.1	1.0
	*S100a6* *				1.3	0.83	*Mt1* *		-0.70			
	*Tnfrsf12a* *					1.2	*S100a6* *	-0.72	1.0	1.0		0.97
	*Vcam1* *					1.6	*Tnfrsf12a* *					1.1
	*Acta2* †			0.76		0.82	*Vcam1* *					1.2
	***Cdkn1a*** †		0.87	1.1	1.5	1.8	*Acta2* †			0.75		0.76
	***Fos*** †					1.0	***Cdkn1a*** †		1.0	1.2	1.5	1.7
	*Ccng1* ‡			0.74		0.93	***Fos*** †			0.62		1.2
	*Cx3cl1* ‡					0.70	*Cxcl9* ‡	0.78				0.72
	*Cxcl12* ‡	0.77					***Pcna*** ‡	0.62				
	*Gadd45g* ‡	0.87					*Tnfrsf21* ‡	-1.0	-1.1			
	*Gjb2* ‡	-0.97		-0.86								
**12 months**	*Acox1* *	-0.73	-0.78	-0.72			*Acox1* *				-0.59	
	*Anxa2* *			0.77	0.72		*Afm* *				-0.68	
	***Anxa3*** *			0.64			*Col4a1* *				0.74	
	*Bcat1* *			0.75			*Lcn2* *				0.91	3.2
	*Col4a1* *			0.68			***Mmp2*** *			0.66	0.70	
	*Cxcl1* *					2.7	*Acta2* †	0.91	0.80	1.1		
	*Egr1* *				0.65		***Cdkn1a*** †	1.3	1.1	1.4	1.1	1.5
	*Gdf15* *			0.70	0.59		***Ccnd1*** ‡				0.58	
	*Hspa1a* *	0.66					*Ccng1* ‡				0.63	
	*Lcn2* *			0.80	0.76	3.6	*Gjb2* ‡	-0.75				
	***Mmp2*** *			0.84	0.93		*Hspe1* ‡				-0.63	
	*Mt1* *	-0.64	-0.70				*Tnfrsf21* ‡		-0.93	-0.64		-1.5
	*S100a6* *	1.3	0.86	1.2	1.0							
	*Vcam1* *			0.88	0.66	2.1						
	*Acta2* †	1.1	0.94	1.1								
	***Cdkn1a*** †	1.3	1.0	1.6	1.4	2.0						
	***Ccnd1*** ‡			0.65	0.61							
	*Gadd45g* ‡		-0.61									
	*Gja1* ‡	0.81			0.79							
	*Gjb2* ‡	-0.59										
	*Lep* ‡				0.83							
	*Tnfrsf21* ‡		-0.91		-1.0							

* Previously proposed kidney damage marker

^†^ Previously proposed kidney damage marker and ionizing radiation marker

^‡^ Previously proposed ionizing radiation marker.

**Bold** indicates that the gene has also been proposed at the protein level to be a potential biodosimeter

**Fig 2:**
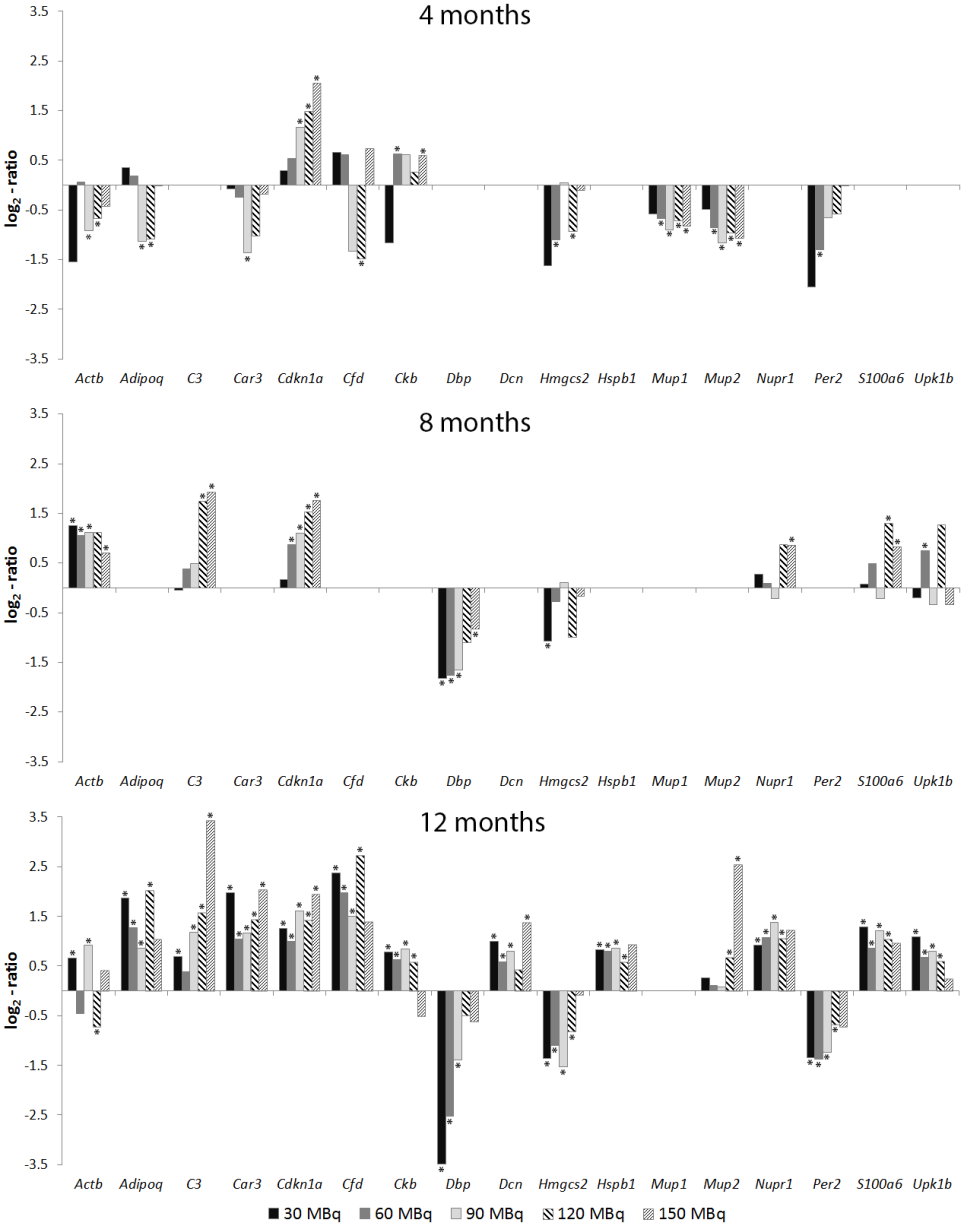
Recurrently regulated transcripts in kidney cortex. Gene expression patterns for differentially regulated transcripts with recurrent expression at one or more time points in kidney cortex 4, 8 or 12 months after administration of 30–150 MBq ^177^Lu-octreotate. * Statistically significant regulation (see Materials and Methods)

**Fig 3:**
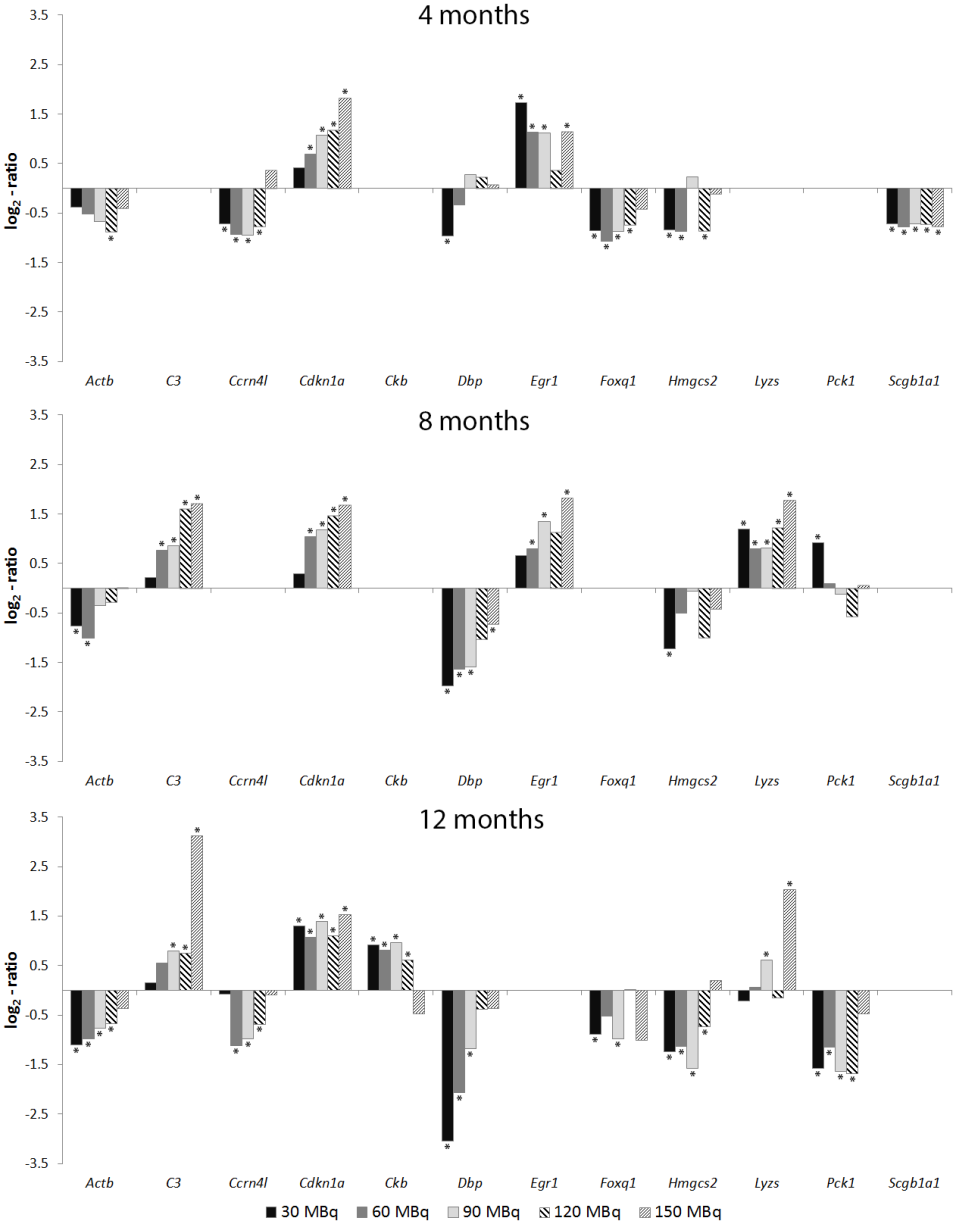
Recurrently regulated transcripts in kidney medulla. Gene expression patterns for differentially regulated transcripts with recurrent expression at one or more time points in kidney medulla 4, 8, or 12 months after administration of 30–150 MBq ^177^Lu-octreotate. * Statistically significant regulation (see Materials and Methods)

Several transcripts were frequently regulated at different time points and absorbed doses (Figs 2 and 3). The *Cdkn1a* (cyclin-dependent kinase inhibitor 1A) gene was differentially regulated at all time-points and the majority of activities for both tissues. The *Erg1* gene was also frequently differentially regulated at the higher activities, with the exception of 120 MBq at all time-points. The *Acta2* gene was frequently observed for the higher activities at 8 months, and for the lower activities at 12 months after injection (30–90 MBq). Transcripts with recurrently altered expression levels, which have not previously been associated with kidney injury or radiation injury, included the *Dbp* gene with altered expression in both tissues for 30–90 MBq at 8 and 12 months. In addition, the *C3* (complement component 3) gene was generally differentially regulated for 90–150 MBq at 8 and 12 months. The *Hmgcs2* (3-hydroxy-3-methylglutaryl-Coenzyme A synthase 2) gene was frequently regulated for 30–120 MBq at 12 months in both tissues.

The frequently regulated transcripts were identified primarily as potential targets of different cytokines and transcription regulators (Table 2). The up-stream regulators *Ifng* and *Tnf* were most strongly associated with the kidney cortex, whereas *Ifng*, *Il1B*, and *Nr3c1* were most strongly associated with the kidney medulla.

**Table 2:** Upstream regulators. Top upstream regulators of transcripts with recurrent expression (cf. Figs 2 and 3) in kidney cortex and medulla

Upstream regulators	Kidney Cortex	Kidney Medulla	Description
***Ifng***	*Actb*, *Adipoq*, *C3*, *Cdkn1a*, *Dbp*, *Hspb1*, *Nupr1*	*Actb*, *C3*, *Cdkn1a*, *Dbp*, *Egr1*, *Pck1*	Interferon gamma, Cytokine
***Tnf***	*Actb*, *Adipoq*, *C3*, *Cdkn1a*, *Cfd*, *Dcn*, *Per2*	*C3*, *Cdkn1a*, *Egr1*	Tumor necrosis factor, Cytokine
***Cebpb***	*Adipoq*, *C3*, *Cdkn1a*, *Cfd*, *Dcn*, *Nupr1*	*C3*, *Cdkn1a*	CCAAT/enhancer binding protein (C/EBP) beta, Transcription regulator
***Il1B***	*C3*, *Cdkn1a*, *Dbp*, *Dcn*, *Hspb1*, *S100a6*	*C3*, *Ccrn4l*, *Cdkn1a*, *Dbp*, *Egr1*	Interleukin 1 beta, Cytokine
***Cebpa***	*Adipoq*, *C3*, *Cdkn1a*, *Cfd*, *Mup1*		CCAAT/enhancer binding protein (C/EBP) alpha, Transcription regulator
***Pparg***	*Adipoq*, *Cdkn1a*, *Cfd*, *Hmgcs2*	*Cdkn1a*, *Hmgcs2*, *Pck1*	Peroxisome proliferator-activated receptor gamma, Ligand-dependent nuclear receptor
***Erbb2***	*Actb*, *Cdkn1a*, *Hspb1*, *S100a6*		Erythroblastic leukemia viral oncogene homolog 2, Kinase
***Hoxa10***	*Actb*, *Adipoq*, *Cdkn1a*, *Cfd*	*Actb*, *Cdkn1a*	Homeobox A10, Transcription regulator
***Ins1***	*Actb*, *Adipoq*, *Cdkn1a*, *Cfd*		Insulin I, Hormone
***Lep***	*Adipoq*, *Cdkn1a*, *Cfd*, *Per2*	*Cdkn1a*, *Pck1*	Leptin, Growth factor
***Psen1***	*Actb*, *C3*, *Ckb*, *Dbp*, *Nupr1*		Presenilin 1, Peptidase
***NFkB (complex)***	*C3*, *Cdkn1a*, *Dbp*, *Hspb1*	*C3*, *Cdkn1a*, *Dbp*, *Egr1*	NFkB (complex)
***Hras***	*Actb*, *Cdkn1a*, *Hspb1*	*Actb*, *Cdkn1a*, *Egr1*, *Pck1*	Harvey rat sarcoma viral oncogene homolog, Enzyme
***Edn1***	*Actb*, *Adipoq*, *Nupr1*	*Egr1*	Endothelin 1, Cytokine
***Egfr***	*Actb*, *Cdkn1a*, *Hspb1*	*Actb*, *Cdkn1a*, *Egr1*	Epidermal growth factor receptor, Kinase
***Nr3c1***		*Actb*, *Cdkn1a*, *Hmgcs2*, *Pck1*	Nuclear receptor subfamily 3 C1, Ligand-dependent nuclear receptor

### Histological evaluation

**Fig 4:**
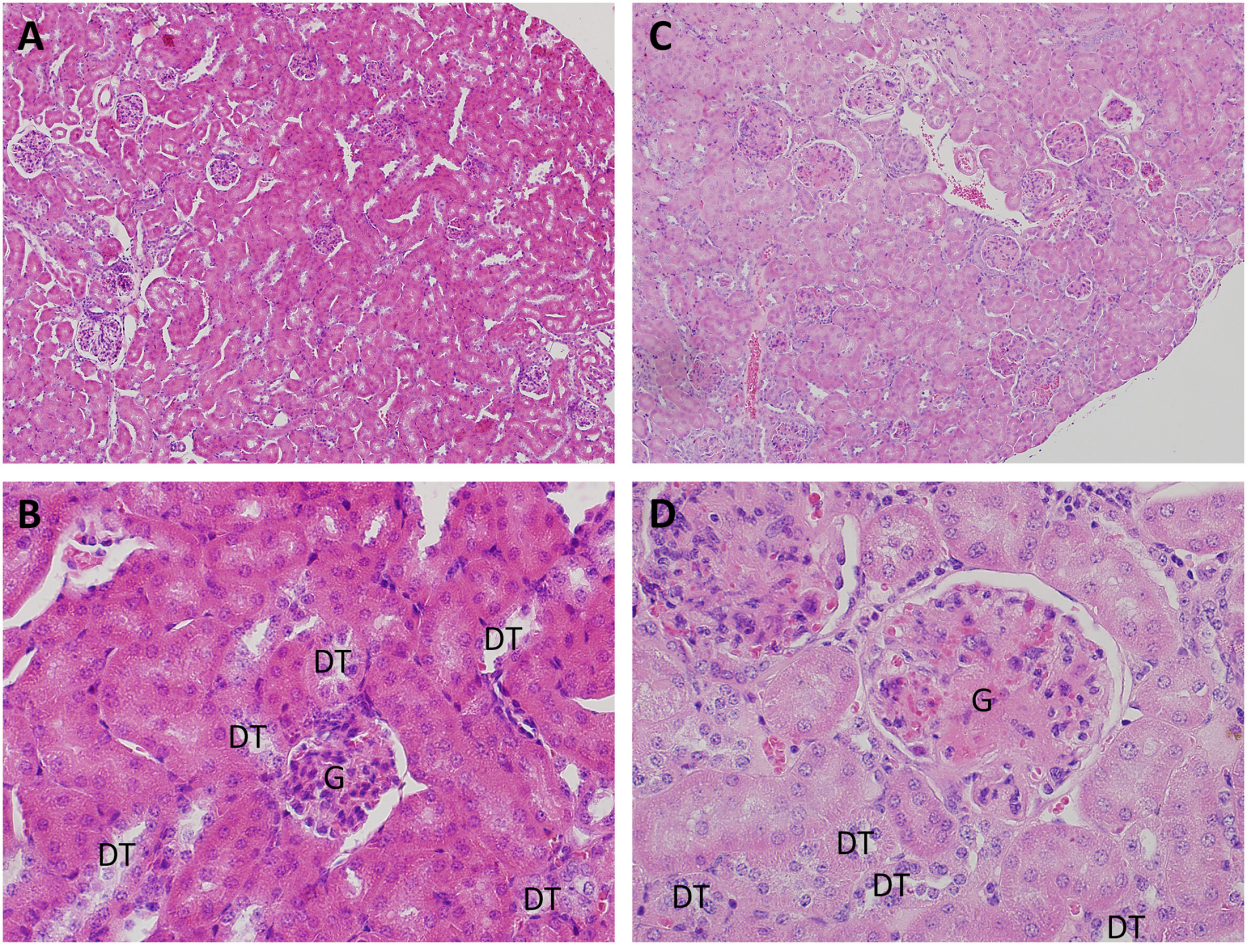
Kidney histology. Kidney histology in control animals as well as in animals where no histological changes were seen at (a) 10x magnification and (b) 40x magnification. The glomeruli are normocellular, with open capillary loops. No signs of segmentalization, necrosis or sclerosis were seen. The tubular, interstitial, and vascular compartments were all without histological changes, no signs of necrosis or inflammation were noted. The histological changes associated with the group receiving 150 MBq at 12 months are shown at (c) 10x magnification and (d) 40x magnification. The changes were localized to the glomeruli. These displayed focal signs of segmental sclerosis and segmentalization of the glomeruli, indicating cellular injury to the mesangium and glomerular capillaries. Edemtatous closure of the capillary loops was noted. Furthermore, the nuclei displayed degenerative changes with polymorphism and nuclear fragmentation, potentially indicating detrimental radiation effects. In a fraction of the glomeruli, signs of segmental fibrinoid necrosis were seen, again underscoring acute injury to the glomeruli. Tubules, interstitium and vasculature showed no signs of injury in these tissues. Staining by standard hematoxylin and eosin. The glomeruli are depicted with”G” and the distal tubules with”DT”.

The histological evaluation revealed glomerular segmentalization and signs of nodular sclerosis after 150 MBq at 8 months after injection (Fig 4). In addition, at 12 months, prominent nucleol and nuclear fragmentation was observed as well as focal and segmental necrosis for the 150 MBq group. No clear observations of kidney injury were observed at the other activity-levels and time-points investigated.

### Scintigraphy

**Fig 5:**
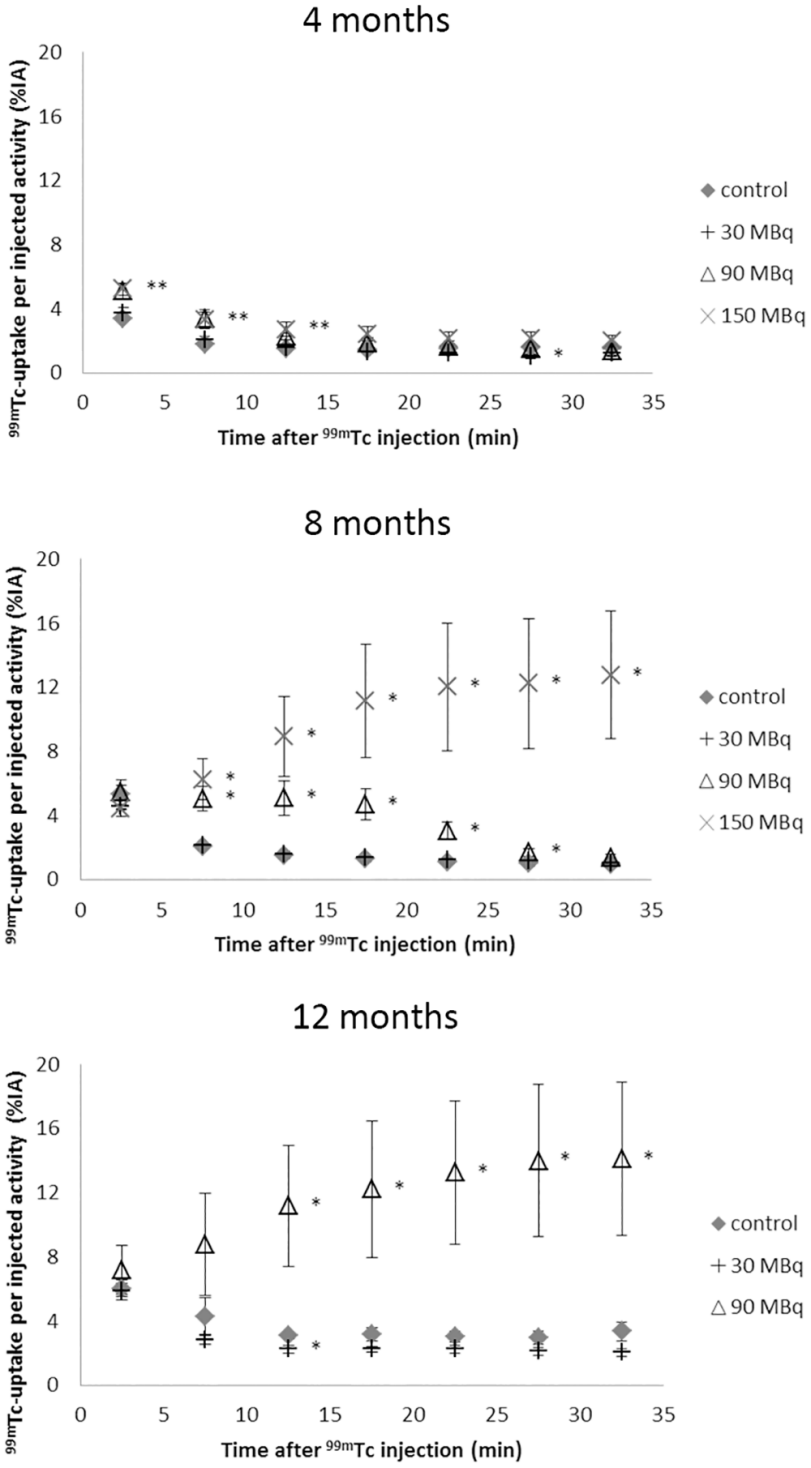
^99m^Tc-DTPA scintigraphy. Results from ^99m^Tc-DTPA scintigraphy performed 4, 8, and 12 months after administration of 0, 30, 90 or 150 MBq ^177^Lu-octreotate. The kidney uptake is presented as percent of injected activity. Error bars represent SEM and * indicates statistically significant difference compared with controls (p <0.05), and at 4 months the ** indicates statistical significance for the 90 and 150 MBq groups.

**Fig 6:**
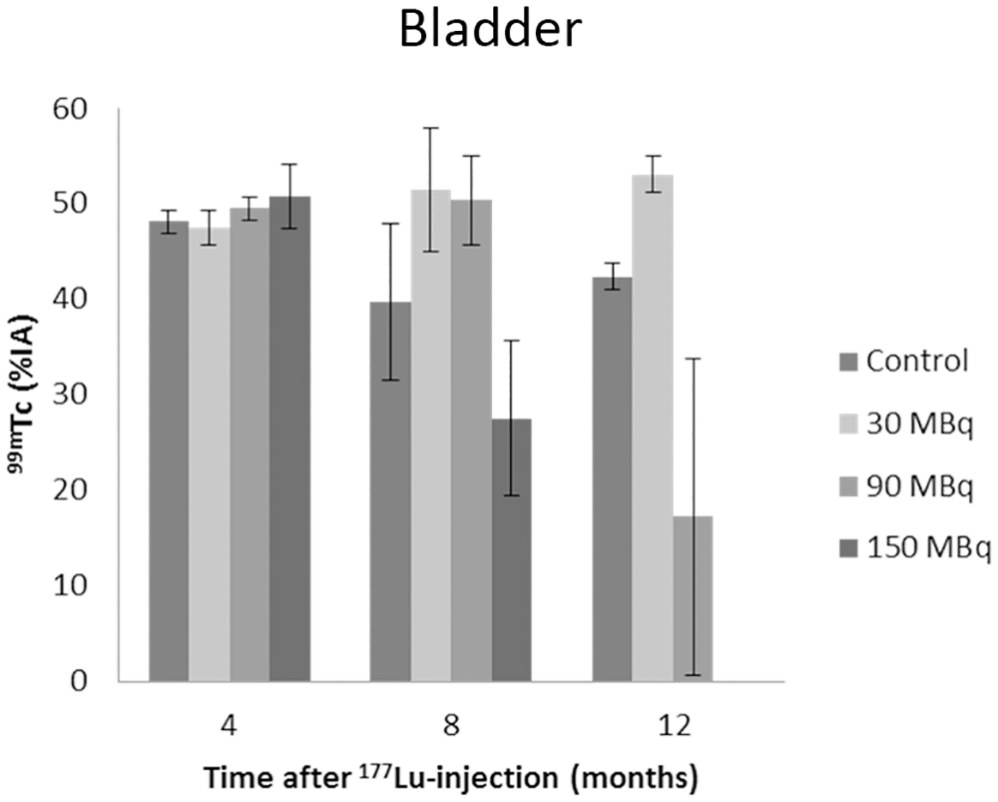
Urinary bladder content following ^99m^Tc-DTPA administration. Urinary bladder content at 27.5 min after ^99m^Tc-DTPA administration. Scintigraphy was performed at 4, 8, and 12 months after administration of 30–150 MBq ^177^Lu-octreotate. Kidney uptake is presented as percent of injected activity. Error bars represent SEM

The time-activity curves for ^99m^Tc-DTPA revealed a statistically significant increased accumulation in the kidneys at 8 and 12 months after ^177^Lu-octreotate administration for the 90 and 150 MBq groups (Fig 5). At 8 months, a slightly increased and delayed retention of ^99m^Tc-DTPA was observed after 90 MBq, while after 150 MBq, ^99m^Tc was accumulated in the kidneys with largely reduced excretion to the urinary bladder (Fig 6). At 12 months, the 90 MBq group showed the same trend of reduction in excretion to the bladder as was shown for 150 MBq at 8 months. Measurements of the 150 MBq group at 12 months were not possible since most of the mice were killed after 8 months due to reduced physical condition. At 4 months, a significantly higher uptake between 2.5 and 12.5 min after ^99m^Tc administration was observed for the 90 and 150 MBq groups. There was no statistically significant difference in ^99m^Tc activity in the kidneys between the 30 MBq group and the control group at any time point, except for two time-points at 4 and 12 months (Fig 5).

**Fig 7:**
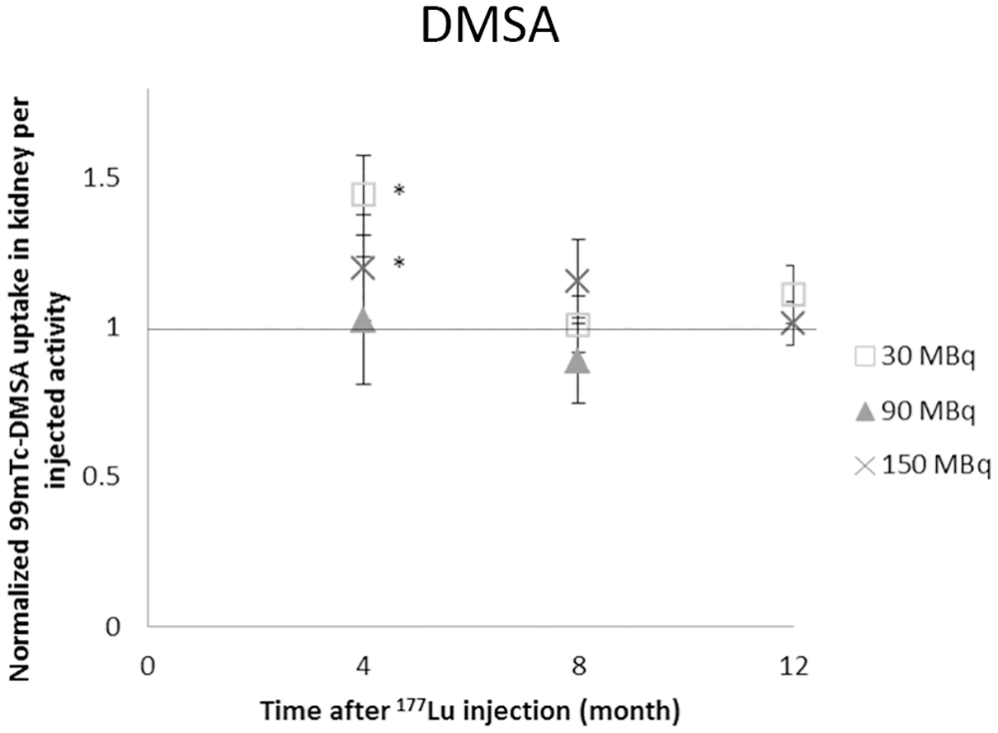
^99m^Tc-DMSA scintigraphy. Results from ^99m^Tc-DMSA scintigraphy performed 4, 8, and 12 months after ^177^Lu-octreotate administration. The kidney uptake is presented as percent of injected activity normalized to controls. Error bars represent SEM and * indicates statistically significant difference compared with controls (p<0.05)

A statistically significant increase in uptake of ^99m^Tc-DMSA compared with controls was observed at 4 months for mice injected with 30 and 150 MBq ^177^Lu-octreotate (p<0.05) (Fig 7). Furthermore, a time-dependent accumulation of the ^99m^Tc was observed, with increased uptake at 8 and 12 months compared with 4 months.

### Blood sample analyses

The mean white blood cell count at 4 months (compared to the control group) was reduced by a factor 2.0, 1.9, and 1.6 after 30, 90, and 150 MBq, respectively (Table 3). No or only minor differences in red blood cell count was detected. Measurements at longer time-points were not possible due to technical difficulties.

**Table 3:** Overview of the results. Overview of the results concerning body weight, blood cell count, urea and creatinine blood levels, ^99m^Tc-DTPA and ^99m^Tc-DMSA scintigraphy, and histology. The results are presented as test divided by control (given as percent)

Days after therapy	IA	Body weight (time of death)	White blood cell count	Red blood cell count	Urea	Creatinine	^99m^Tc-DTPA*	^99m^Tc-DMSA	Histology
**4 months**	**30 MBq**	110%	50%	99%	130%	100%	66% (27.5 min)	140%	Normal
	**60 MBq**	98%	ND	ND	97%	100%	ND	ND	Normal
	**90 MBq**	110%	53%	94%	91%	100%	87% (32.5 min)	100%	Normal
	**120 MBq**	99%	ND	ND	92%	100%	ND	ND	Normal
	**150 MBq**	89%	63%	95%	91%	100%	59% (32.5 min)	120%	Normal
**8 months**	**30 MBq**	90%	ND	ND	230%	100%	62% (32.5 min)	100%	Normal
	**60 MBq**	110%	ND	ND	140%	100%	ND	ND	Normal
	**90 MBq**	100%	ND	ND	140%	100%	270% (12.5 min)	89%	Normal
	**120 MBq**	100%	ND	ND	280%	100%	ND	ND	Normal
	**150 MBq**	85%	ND	ND	280%	100%	450% (27.5 min)	110%	Abnormal
**12 months**	**30 MBq**	98%	ND	ND	110%	100%	160% (17.5 min)	110%	Normal
	**60 MBq**	120%	ND	ND	82%	100%	ND	ND	Normal
	**90 MBq**	110%	ND	ND	140%	100%	910% (27.5 min)	ND	Normal
	**120 MBq**	110%	ND	ND	160%	100%	ND	ND	Normal
	**150 MBq**	110%	ND	ND	520%	110%	ND	100%	Abnormal

*Values in parenthesis indicates time after ^99m^Tc-DTPA injection where highest deviation from control was observed

ND = not done

The creatinine levels in blood revealed no or only minor differences compared with controls. A 5-fold increase in blood urea levels were found at 12 months after 150 MBq (p<0.05), whereas lower values were observed at the other time points and activity levels.

## Discussion

The present study represents a comprehensive investigation of the long-term transcriptional, functional, and morphological effects of ^177^Lu-octreotate administration on kidney tissue, one of the main limiting organs in ^177^Lu-octreotate therapy. Distinct late toxic effects were seen in mice, with reduced kidney function, demonstrated by scintigraphy, morphology, and urea level. Furthermore, the transcriptional analyses revealed a distinct dose-dependent regulation of expression, which correlated with the functional and morphological data.

In the current study, the absorbed dose was calculated to whole kidney. The distribution of the radionuclides in the kidneys is heterogeneous, and previous studies have shown a higher uptake in the kidney cortex compared to medulla at early time points after injection (24h) [8, 37], with concentrations of at least 50–60% in the outer medulla compared to cortex. However, gender dependencies have also been found, where female mice showed higher uptake in the outer medulla compared with the cortex [37]. Due to these discrepancies, and possible mouse strain dependencies, no more detailed tissue dosimetry in the kidneys was performed. Furthermore, the number of animals in the present study was limited to 3–4 per group. While a higher number of animals per group would result in a stronger power to the analysis, the similar genotype of this inbred mouse strain (C57BL/6N) together with tightly controlled working conditions have previously shown a strong reproducibility of the results with 3 animals per group. Unpublished data from our group has also shown that the similarity between the animals in gene expression at the current cut offs used was between 60 and 86%. Due to these facts and ethical considerations, three animals per group were chosen for the transcriptional response analysis.

Several of the differentially regulated transcripts identified in the present study have previously been proposed as biomarkers of radiation damage and kidney injury [19, 33–35, 38–40]. In the present study *Cdkn1a* gene was differentially regulated in 12 of the 15 analyzed groups in kidney cortex, and 13 of the 15 analyzed groups in kidney medulla. The *Cdkn1a* gene is a cycline dependent kinase inhibitor highly involved in cell cycle progression and has previously been shown to be consistently regulated in response to ionizing radiation [33–35]. The *Cdkn1a* gene has also been found to be up-regulated shortly after acute renal failure [38]. In the present study, a dose-response relationship was found for both kidney cortex and medulla at all the time-points studied, with increased transcriptional levels with increased absorbed dose. The transcriptional levels for the *C3* (complement component 3) and *Dbp* (albumin promoter binding protein) genes also exhibited an absorbed dose dependence for both tissues, whereas the *Per2* (periodic circadian clock 2) and *Actb* (actin, beta) genes showed a dose-response relationship in kidney cortex and medulla, respectively. These genes have not previously been associated with radiation damage or kidney induced injury, which may indicate the specificity of these genes in the response to administration of specific radiopharmaceuticals.

The pathway analysis of the recurrently regulated transcripts predicted the *Ifng* and *Tnf* genes in kidney cortex, and the *Ifng*, *Il1B*, and *Nr3c1* genes in kidney medulla as the most highly associated up-stream regulators. The *Ifng*, *Tnfa*, and *Il1* genes have been shown to be involved in “danger” signaling following irradiation, with guidance of orchestration of tissue repair [41]. These genes are also involved in cell cycle arrest, cell survival, DNA repair, and senescence [41]. The *Nfkb* complex was also identified as an up-stream regulator, which have been found to be one of the most frequently activated transcription factors by radiation exposure [41–43]. Taken together, these data indicate the importance of transcription factors and cytokine regulation in the response to kidney exposure to ^177^Lu-octreotate.

An ideal biomarker for radiation-induced kidney injury should preferably be organ specific and originate from the damaged cells. The dose-response relationship of the biomarker should be directly dependent on the extent of damage, and the change in transcriptional expression should be expressed early after insult [44]. These criteria are of the highest concern to optimize individualized patient treatment. Several of the genes in the present study have also been previously found to be differentially expressed at earlier time points than the 4–12 months studied here. The *Car3*, *Cdkn1a*, *Dbp*, *Hmgcs2*, *Mup2*, *Per2*, *S100a6*, and *Upk1b* genes and the *Actb*, *Adipoq*, *Car3*, *Cdkn1a*, *Cfd*, *Ckb*, *Dbp*, *Hmgcs2*, *Nupr1*, *Per2*, and *Upk1b* genes were differentially regulated at 24h after ^177^Lu-octreotate administration in the kidney cortex and medulla, respectively [31]. The *Cdkn1a* gene showed increased expression with increased absorbed dose also at this early time point in both kidney cortex and medulla. The *Dbp* and *Hmgcs2* genes were differentially regulated at all dose levels studied (0.13–13 Gy) in both tissues, and the *Per2* gene was differentially regulated at absorbed doses between 0.34 and 13 Gy. Already at this early time point (24h after administration), the consistent change in transcriptional levels for several of these genes indicated their potential utility as biomarkers of internal exposure to radionuclides and renal tissue injury.

The histological evaluation revealed clear signs of kidney toxicity at the highest activity level investigated (150 MBq, 54 Gy) at 8 and 12 month after administration. The effects in the present study concerned the glomeruli with sclerosing and segmentalization (injury of glomerular capillaries and mesangium) of the glomerular tuft. In addition, at 12 months, signs of cell death could also be observed in the glomeruli in the form of nuclear fragmentation and segmental necrosis. In addition, signs of inflammation were seen. No changes regarding tubules, interstitium or other vessels were observed. These results are in contrast to previously published morphological effects of ^177^Lu-octreotate on rats (46–92 Gy, 109 days after injection) and nude mice (35–58 Gy, 6 months after injection) [45, 46], demonstrating mainly tubulointerstitial changes after ^177^Lu-octreotate administration. These consisted of inhomogeneous nuclei formation, apoptosis, and necrotic cells in the proximal and distal tubules as well as flattening of tubular epithelium, loss of brush border, dilation, and empty lumina in a dose-dependent manner after ^177^Lu-octreotate administration, respectively. In contrast, no or only very mild changes of the glomeruli was observed in these studies.

^99m^Tc-DTPA scintigraphy clearly revealed changes in kidney function with increased amount of ^177^Lu-octreotate administered and with time after administration. At 4 months after injection no difference in filtration or excretion rate could be observed in any of the groups. At 8 months, an altered time-activity curve was observed for the 90 MBq group, while for the 150 MBq group an accumulation of ^99m^Tc in the kidneys was observed with no or altered emptying into the bladder. At 12 months, few of the animals in the 150 MBq group were alive. The reduced excretion of ^99m^Tc for the 90 MBq group at 12 months was similar to that observed for the 150 MBq group at 8 months, indicating a longer time until renal function loss at reduced activity. No effects on renal clearance curves were observed for the 30 MBq group at any time-point.

In general, the time-activity curve of the kidneys after ^99m^Tc-DTPA administration shows a rapid uptake phase for 1–3 minutes followed by a slow excretion phase when ^99m^Tc is transported to the bladder [14]. The slope of the uptake phase is correlated with GFR. However, due to practical issues, the initial uptake-phase could not be measured in the present study, and the scintigraphic data represent the excretion of ^99m^Tc-DTPA. In a previous study on rats using ^111^In-DTPA to measure renal function 100–120 days after administration of 460 MBq ^177^Lu-octreotate, a reduced maximal uptake of ^111^In in the kidneys compared with untreated controls was found [15]. In the present study we did not measure the maximal uptake due to technical issues, but found a reduced excretion from the kidneys in the most exposed groups. To our knowledge, a rise in the excretion phase of the renal time-activity-curve of ^99m^Tc-DTPA has not been previously observed after ^177^Lu-octreotate administration. However, one reason behind such results from ^99m^Tc-DTPA scintigraphy is nephrolithiasis [47]. Calculi in kidneys or ureters may develop from metabolic imbalance in electrolytes. In the present study, a strong association with sodium, calcium, and potassium ion binding was observed among the differentially regulated genes. This observation was most prominent at later time points (8–12 months) at the higher absorbed doses delivered (40–54 Gy), which could imply an electrolyte imbalance in the kidney tissues.

^99m^Tc-DMSA scintigraphy revealed no reduction in uptake for any of the groups. Instead a significant increase in ^99m^Tc-uptake was observed at 4 months after 30 and 150 MBq. The kidney uptake of ^99m^Tc-DMSA after ^177^Lu-octreotate administration has previously been studied in rats [15, 45]. A clear dose-dependent decrease in ^99m^Tc-DMSA uptake was then observed 109–146 days after administration of 278 and 555 MBq ^177^Lu-octreotate (9.9, and 1.4%IA, respectively, vs. 23%IA in control), indicating tubular damage after the highest injected activity [45]. Furthermore, increased ^99m^Tc accumulation was found in the outer medulla with increased injected activity [45]. In the present study, no statistically significant reduction in uptake was observed at any time-point or ^177^Lu activity level investigated, despite the functional obstruction observed with ^99m^Tc-DTPA. However, renal or ureter calculi might reduce the ^99m^Tc-DMSA excretion from the kidneys and hide an otherwise reduced kidney uptake [48].

No functional effects were found in the 16 Gy group, but were clearly evident in the 40 Gy group. Thus, the tolerance dose in mice after a single administration of ^177^Lu-octreotate would be found in the range 16–40 Gy, which is within the range previously reported for both mice and humans [26, 46]. It should be noted that the kidneys would most probably tolerate a higher exposure after fractionated therapy. Furthermore, it is interesting to note that no altered levels of creatinine were detected and clear histological manifestations were only seen at the highest absorbed dose (54 Gy) at 8 and 12 months after administration. This would indicate a possible higher tolerance dose for the kidneys in these mice, compared to the radiosensitive Balb/c mice previously used for toxicity assessments [46]. Furthermore, a distinct dose response relationship was observed for several of the regulated transcripts of, e.g. the *Cdkn1a*, *Dbp*, and *Per2* genes. Together with the functional morphological data, these transcriptional variations were correlated to functional impairment and injury. In a previous study, these transcriptional variations were observed already at 24h after injection of ^177^Lu-octreotate [31]. Thus, altered regulation of these genes may serve as biomarkers that might potentially predict injury already at early time points and the ability to follow the progression of the induced injury. However, further studies are needed to establish this correlation. In addition, the protein expression levels of these genes in kidney tissues, blood, and urine also need to be studied to establish their clinical utility.

### Conclusions

In the present study, a comprehensive investigation of the transcriptional, functional, and morphological effects of administration of high amounts of ^177^Lu-octreotate on normal kidney tissue was performed. A strong and diverse transcriptional response was observed, and the functional investigations revealed clear negative effects on renal function, with enhanced effects with absorbed dose and time after administration. Furthermore, the gene expression studies revealed several potentially useful biomarkers, e.g. *Cdkn1a*, *C3*, *Dbp*, *Lcn2*, and *Per2*, that might be used as early predictors of late renal injury. Further studies are needed to evaluate their clinical usefulness.
